# Determination of hemolysis index thresholds for biochemical tests on Siemens Advia 2400 chemistry analyzer

**DOI:** 10.1002/jcla.22856

**Published:** 2019-02-19

**Authors:** Zhenhua Du, JiQin Liu, Hua Zhang, BuHe Bao, RuiQi Zhao, Ying Jin

**Affiliations:** ^1^ Department of clinical laboratory, Characteristic Medical center of Chinese People’s Armed Police Force Pingjin Hospital Tianjin China

**Keywords:** biochemical test, hemolysis index, interference, laboratory medicine

## Abstract

**Background:**

In vitro hemolysis is still the most common source of pre‐analytical nonconformities. This study aimed to investigate the hemolytic effects on commonly used biochemical tests as well as to determine the hemolysis index (HI) thresholds on Siemens Advia 2400 chemistry analyzer.

**Methods:**

Peripheral blood samples were collected from forty healthy volunteers. Hemolysis was achieved using syringes. Five hemolysis levels were produced including the no hemolysis group, slight hemolysis group, mild hemolysis group, moderate hemolysis group, and heavy hemolysis group. We then used the bias from baseline (no hemolysis) and HI to construct regression functions. The HI corresponding to the bias limits was considered as HI thresholds. We chose the total allowable error (TAE) as the bias limit.

**Results:**

Of the twenty‐eight analytes, ten analytes had clinical significance. Creatine kinase‐MB, creatine kinase, potassium, aspartate aminotransferase, and hydroxybutyrate dehydrogenase were all positively affected; the corresponding HI threshold was 45.2, 99.96, 4.07, 10.16, and 7.94, respectively. Lactate dehydrogenase was also positively interfered, but we failed to calculate the HI threshold. Total bile acid, uric acid, and sodium were all negatively affected, and the HI threshold was 42.23, 500 and 501.8, respectively. Glucose was also negatively interfered, but it failed to achieve the HI threshold.

**Conclusions:**

When the HI value was higher than its threshold, the corresponding analyte was considered inappropriate for reporting. The implementation of the assay‐specific HI thresholds could provide an accurate method to identify analytes interfered by hemolysis, which would improve clinical interpretations and further boost laboratory quality by reducing errors associated with hemolysis.

## 1 INTRODUCTION

In vitro hemolysis is the leading source of pre‐analytical nonconformities.^1^, ^2^, ^3^, ^4^ It may lead to erroneous results, which potentially affects the interpretation of laboratory test results, and therefore, it can ultimately influence patient care.^5^ It is reported that in vitro hemolysis specimens account for about 3.3% of blood specimens sent to biochemistry laboratories.^6^ Hemolytic causes include troublesome venipuncture(s), use of inappropriate blood collection devices, and inappropriate handling and transportation of blood tubes.^2^, ^7^ Traditionally, hemolysis is detected by visual detection, but this method is time‐intensive, arbitrary, and rather subjective, which consequently impact clinical decisions.^8^ Moreover, it is difficult to visually detect subtle differences in color between hemolysis and icteric samples. The continuous‐flow automatic system leaves little chance for visual detection, and it has been reported that intravenous catheters and vacuum blood‐drawing technology might result in a higher risk of hemolysis;^9^ therefore, the increasing use of these technologies makes it more challenging to quickly identify hemolysis specimens.

Hemolysis index (HI) generated by analyzers is an effective tool to counteract the hemolysis challenge, as it can standardize the process of identifying hemolytic specimens and estimate the hemolysis interferences quantitatively.^10^, ^11^, ^12^, ^13^, ^14^, ^15^, ^16^, ^17^ Even though it was reported that the hemolysis index (HI) was accurate and highly reproducible among different platforms and laboratories,^16^ determining the HI threshold is the key to identify hemolytic effects. In particular, as different analytical platforms have various assay parameters, using one set of HI thresholds across all platforms is impossible. Given the lack of studies specified in HI thresholds on Siemens Advia 2400 chemistry analyzer, we aimed to investigate HI thresholds on a Siemens Advia 2400 chemistry analyzer according to the total allowable error (TAE).^18^, ^19^

## 2 METHODS

### 2.1 Subjects and methods

Peripheral venous blood samples were collected from 40 healthy volunteers. We obtained approval from the institutional ethics committee. Informed consent was obtained from participants according to the committees’ regulations. 8 mL of venous blood was drawn from each of the participants and transferred into two 4‐mL tubes coated with lithium heparin (CXQ004, 13 × 100 mm, Shenzhen Boon Medical Supply Co., Ltd., Shenzhen, China). Subsequently, they were split into 5 heparinized tubes (CXQ004, 13 × 100 mm, 3 mL, Shenzhen Boon Medical Supply Co., Ltd., Shenzhen, China). After which, we collected five sets of samples. Samples belonging to one set were labeled as baseline samples without hemolysis, while samples in the other four sets were hemolyzed by mechanical trauma to obtain increasing degrees of hemolysis, as previously reported.^12^, ^13^, ^20^ The four sets of samples were respectively aspirated 2, 4, 6, and 8 times through needles attached to 5‐mL syringes (1.5 inch, 21 gauge) to produce slightly, mildly, moderately, and heavily hemolyzed samples. They were all subsequently centrifuged at 1000 g for 15 minutes. The Advia 2400 (Siemens Healthcare Diagnostics Inc, Deerfield, IL, USA) measured serum/plasma absorbance at 571 and 596 nm for hemolysis (ABS_H), and 658 and 694 nm for lipemia index (ABS_L), respectively. It then reported the HI using the formula: HI=3942.6×(ABS_H‐1.156 × ABS_L). The HI value was then compared to the qualitative judgment set and flag samples when appropriate, including no hemolysis (−) for HI <23, slight hemolysis (+) for 23 ≤ HI<110, mild hemolysis (++) for 110 ≤ HI<234, moderate hemolysis (+++) for 234 ≤ HI<379, and heavy hemolysis (++++) for HI≥379.

Plasma concentrations of alanine aminotransferase (ALT), aspartate aminotransferase (AST), total bile acid (TBA), total protein (TP), albumin (ALB), total bilirubin (TBIL), direct bilirubin (DBIL), alkaline phosphatase (ALP), lactate dehydrogenase (LDH), gamma‐glutamyltransferase (GGT), creatine kinase (CK), creatine kinase‐MB (CKMB), hydroxybutyrate dehydrogenase (HBDH), glucose (GLU), amylase (AMY), uric acid (UA), total cholesterol (CHOL), triglyceride (TG), high‐density lipoprotein cholesterol (HDL‐C), low‐density lipoprotein cholesterol (LDL‐C), apolipoprotein A1 (APO‐A1), apolipoprotein B (APO‐B), calcium (Ca), iron (Fe), potassium (K), sodium (Na), phosphate (P), and lipoprotein(a) (Lp(a)) were analyzed in the Siemens Advia 2400 chemistry analyzer according to the corresponding reagent protocols. The assay reagents were obtained from the same vendor as the analyzer system (Siemens Healthcare Diagnostics Inc, Deerfield, IL, USA). The hemoglobin (Hb) level was measured on an XE‐5000 hematology analyzer (Sysmex Corporation, Kobe, Japan).

### 2.2 Statistical analysis

All statistical analyses were carried out using the Statistical Package for the Social Sciences 19.0 (SPSS Inc, Chicago, IL, USA) and GraphPad Prism 7 (GraphPad Software Inc, USA). Normality of the data was investigated by Kolmogorov‐Smirnov test. Continuous variables were expressed as the mean ±standard deviation (SD), whereas categorical variables were expressed as numbers (percentages). Spearman correlation was performed to investigate the relationship between HI and hemoglobin concentrations. The positive or negative change (bias) in the analyte concentration was determined using the formula: bias% = 100×(concentration in hemolysis sample – concentration in baseline sample)/concentration in baseline sample. The Clinical Laboratory Improvement Amendments of 1988 (CLIA'88) established TAE for assessing methods and laboratory performance for specific regulated analytes.^18^ We chose ±10% as maximum allowable bias for TBA, LDL‐C, APO‐A1, APO‐B, GGT, HBDH, P, and Lp(a), as there are no given acceptable limits in CLIA’88 for them.^19^ The 2‐tailed t test was used to compare analyte concentrations between hemolysis groups and the baseline group (no hemolysis). These results revealed a higher bias than TAE limits, and statistical differences from baseline concentrations were considered to be clinically significantly interfered by hemolysis. In order to identify HI thresholds for interference analysts, we used the “curve estimation” in SPSS including linear, logarithmic, inverse, quadratic, and cubic models to select the model with the highest R2 and the lowest P values. We then used the GraphPad Prism 7 to produce graph and formula of regression curves chosen from the curve estimation, through which we could precisely locate the x (HI) and y (bias) coordinates on the curves. The HI corresponding to the bias limits (TAE or ±10%) was considered as the HI threshold. A P value of <0.05 was considered statistically significant.

## 3 RESULTS

Samples were grouped into five groups according to their hemolysis index flags recommended by the manufacturer, which included the no hemolysis group (NH, n = 40), slight hemolysis group (SH, n = 28), mild hemolysis group (LH, n = 26), moderate hemolysis group (MH, n = 13), and heavy hemolysis group (HH, n = 14), as shown in Table 1. At first, the Hb concentrations in the five groups were measured to evaluate the relationship between HI and Hb. The HI values among the five groups were significantly different when compared to each other (*P* < 0.05); there was a strong association between HI and Hb concentrations (Table 1, *r* = 0.982, *P* < 0.05, Supplement Figure S1). In baseline samples, the Hb concentrations were <0.5 g/mL; both TBIL (10.93 ± 2.7 umol/L）and TG (1.02 ± 0.51 mmol/L) concentrations were less than the corresponding lower reference limits.

**Table 1:** Mean ± SD of the analytes, HI and hemoglobin concentrations for each group, %bias from NH for analytes, ±acceptable limits from CLIA’88, and these results with higher bias than TAE limits, and statistically significant differences compared with NH are marked in bold

	NH(n = 40)	TAE	SH (n = 28) +	LH (n = 26) ++	MH (n = 13) +++	HH (n = 14) ++++
Hemolysis index	7.3 ± 6.39		57.2 ± 25.2*	145 ± 29.1*	284 ± 34.4*	634 ± 188*
Hemoglobin (g/L)	<0.5		1 ± 0.36	1.83 ± 0.27	3.25 ± 0.64	5.54 ± 1.46
Alanine aminotransferase (U/L)	13.7 ± 5.51		15.7 ± 5.83	13.6 ± 5.1	13.54 ± 5.06	14.9 ± 5.82
		±20%	14.6%	−0.73%	−1.17%	8.76%
Aspartate aminotransferase (U/L)	18.0 ± 3.72		30.5 ± 5.2*	37.4 ± 9.41*	46.1 ± 9.58*	71.2 ± 12.6*
		±20%	**69.4%**	**108%**	**156%**	**296%**
Total bile acid (μmol/L)	2.90 ± 1.68		2.56 ± 1.74	1.68 ± 1.6*	1.32 ± 1.85*	0.22 ± 1.85*
		±10%^a^	−11.7%	**−42.1%**	−**54.5%**	**−92.4%**
Total protein (g/L)	73.0 ± 4.25		74.6 ± 4.78	74.9 ± 4.04	73.9 ± 3.47	75.7 ± 3.29
		±10%	2.19%	2.60%	1.23%	3.70%
Albumin (g/L)	42.6 ± 2.64		43.5 ± 3.09	43.9 ± 2.17	43.0 ± 1.55	44.9 ± 1.63*
		±10%	2.11%	3.05%	0.94%	5.40%
Total bilirubin (umol/L)	10.9 ± 2.70		11.0 ± 2.55	11.1 ± 2.88	11.8 ± 2.67	12.8 ± 2.75
		±20%	0.92%	1.83%	8.26%	17.43%
Direct bilirubin (μmol/L)	3.07 ± 1.32		3.70 ± 1.20	3.04 ± 0.87	3.05 ± 0.7	3.7 ± 0.72
		±10%^a^	20.5%	‐0.98%	‐0.65%	20.5%
Alkaline phosphatase (U/L)	63.6 ± 26.9		68.8 ± 41. 9	66.4 ± 20.8	65.46 ± 23.9	67.6 ± 25.0
		±30%	8.18%	4.40%	2.92%	6.29%
Lactate dehydrogenase (U/L)	189 ± 41.9		463 ± 105*	639 ± 168*	841 ± 194*	1308 ± 238*
		±20%	**145%**	**2385%**	**345%**	**592%**
Gamma‐glutamyltransferase (U/L)	17.0 ± 8.07		18.7 ± 13.1	13.8 ± 5.04	12.2 ± 3.08	14.2 ± 2.99
		±10%^a^	10.0%	−18.8%	−28.2%	−16.5%
Creatine kinase (U/L)	87.9 ± 42.0		106 ± 27.9	122 ± 52.6*	138.6 ± 38.5*	193 ± 51.6*
		±30%	20.592%	**38.8%**	**57.7%**	**119%**
Creatine kinase‐MB b(U/L)	7.75 ± 3.86		25 ± 8.95*	43.9 ± 11.8*	76.4 ± 16.0*	150 ± 28.8*
		±3 s (11.58 U/L)	**17.2**	**36.1**	**68.6**	**142**
Hydroxybutyrate dehydrogenase (U/L)	153 ± 30.5		356 ± 87.4*	515 ± 135*	686 ± 148*	1072 ± 155*
		±30%	**133%**	**237%**	**348%**	**601%**
Glucose (mmol/L)	3.15 ± 1.8		2.07 ± 2.72	1.38 ± 0.98*	2.06 ± 0.66	1.79 ± 0.69*
		±10%	−34.3%	−**56.2%**	−34.6%	−**43.2%**
Amylase (U/L)	53.15 ± 14.96		46.2 ± 13.3	56.3 ± 14.9	56.9 ± 15.8	52.2 ± 14.4
		±30%	−13.0%	5.93%	7.06%	−1.79%
Uric acid (μmol/L)	273 ± 58.1		283 ± 64.5	249 ± 55.0	230 ± 51.4	222 ± 47.9*
		±17%	3.66%	−8.79%	−15.7%	−**18.7%**
Total cholesterol (mmol/L)	4.27 ± 0.8		4.02 ± 0.91	4.29 ± 0.71	4.23 ± 0.87	4.28 ± 0.77
		±10%	−5.86%	0.47%	−0.94%	0.23%
Triglyceride (mmol/L)	1.02 ± 0.51		1.18 ± 0.71	0.97 ± 0.4	0.95 ± 0.41	1.04 ± 0.4
		±25%	15.7%	−4.90%	‐6.86%	1.96%
High‐density lipoprotein (mmol/L)	1.1 ± 0.23		1.05 ± 0.25	1.07 ± 0.17	1.05 ± 0.18	1.06 ± 0.19
		±30%	−4.54%	−2.73%	−4.55%	−3.64%
Low‐density lipoprotein (mmol/L)	2.45 ± 0.7		2.17 ± 0.85	2.43 ± 0.57	2.44 ± 0.77	2.39 ± 0.66
		±10%^a^	−11.4%	−0.82%	−0.41%	−2.45%
Apolipoprotein A1 (g/L)	1.33 ± 0.14		1.31 ± 0.11	1.24 ± 0.23	1.29 ± 0.13	1.28 ± 0.13
		±10%^a^	−1.50%	−6.77%	−3.01%	−3.76%
Apolipoprotein B (g/L)	0.75 ± 0.17		0.69 ± 0.19	0.76 ± 0.15	0.91 ± 0.74	0.69 ± 0.16
		±10%^a^	−8%	1.33%	21.3%	−8%
Calcium b(mmol/L)	2.2 ± 0.1		2.21 ± 0.07	2.22 ± 0.1	2.22 ± 0.11	2.18 ± 0.09
		±0.25 mmol/L	0.01	0.02	0.02	−0.02
Phosphate (mmol/L)	0.91 ± 0.14		1.11 ± 0.23	1.16 ± 0.72	0.88 ± 0.13	0.9 ± 0.14
		±10%^a^	22.0%	27.5%	−3.30%	−1.10%
Iron (μmol/L)	14.7 ± 5.83		15.2 ± 6.41	15.4 ± 7.42	13.5 ± 5.65	18.3 ± 7.9
		±20%	3.40%	4.76%	‐8.16%	24.5%
Potassium b(mmol/L)	4.24 ± 0.81		6.16 ± 1.36*	5.53 ± 1.46*	5.76 ± 1.12*	7.29 ± 0.81*
		±0.5 mmol/L	**1.92**	**1.29**	**1.52**	**3.05**
Sodium b(mmol/L)	147 ± 2.36		148 ± 1.67	147 ± 2.65	144 ± 2.29*	142 ± 1.81*
		±4 mmol/L	1	0	−3	−5
Lipoprotein(a) (mg/L)	200 ± 257		188 ± 211	184 ± 253	209 ± 345	251 ± 365
		±10%^a^	−6%	−8%	4.5%	25.5%

HH(++++), heavy hemolysis group; LH(++), mild hemolysis group; MH(+++), moderate hemolysis group; NH(‐), no hemolysis group; SH(+), slight hemolysis group; TAE, total allowed error recommended by CLIA’88 regulations.

The total allowed error for HBDH was assumed to be 30% according to experience.

a ±10% bias was set as the accepted TAE for the analytes because they are not included in the CLIA’88 regulations.

b To compare with the TAE defined as ERROR in CLIA’88, the hemolysis effects on Ca, K, Na, and CK‐MB are expressed as bias directly.

Significant differences exist among the five groups when compared with each other (*P* < 0.05).

**Figure 1:**
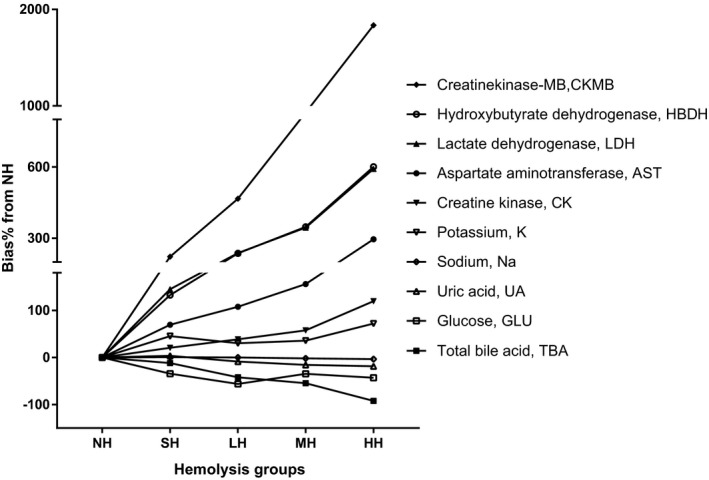
Interferogram for hemolysis and the selected analytes. Y‐axis: %bias of analyte concentrations in comparison with the nonhemolysis group (NH); X‐axis: sample groups with different level hemolysis; lines in the graph represent different analyst. HH, heavy hemolysis group; LH, mild hemolysis group; MH, moderate hemolysis group; SH, slight hemolysis group

We then compared analyte concentrations between hemolysis groups and the baseline group. The concentrations of AST, TBA, ALB, LDH, CK, CKMB, HBDH, GLU, UA, K, and Na in hemolysis groups were significantly different from that of the NH (Table 1, Figure 1, *P* < 0.05). In SH, LH, MH, and HH, the concentrations of AST, LDH, CKMB, HBDH, and K were all higher than that in the NH (*P* < 0.05). In LH, MH, and HH groups, the CK concentrations were greater than the NH group (*P* < 0.05), whereas the TBA concentrations were lower than the NH group (*P* < 0.05). In MH and HH groups, the Na concentrations were fewer than the NH group (*P* < 0.05). In HH, the ALB concentration increased (*P* < 0.05) while the UA concentration decreased (*P* < 0.05), when compared with the NH group. The GLU concentrations decreased significantly only in the LH and HH groups (*P* < 0.05, Table 1).

Next, the biases of the eleven analytes were compared with their CLIA’88 TAE limits^18^ or the 10% bias limits (Table 1, Figure 1, Figure S2). The ALB biases were within the TAE limit in all four hemolysis groups. The biases of AST, LDH, HBDH, CKMB, and K gradually increased relative to their TAE limits in SH (Table 1). The CK biases increased greater than the 30% limit in LH. The TBA biases became lower than the −10% limits in LH. The biases of UA became lower than the −17% limits in HH. The biases of Na became lower than the −4 mmol/L limit in MH. The biases of GLU were lower than the −10% limit only in the LH and HH groups.

The regression analysis was eventually employed to model the relationship between bias and HI for these interfered analytes (Table 2, Figure S2). Because there was no relationship between HI and bias for GLU (*P* > 0.05), we failed to obtain the model for GLU. The HI threshold for AST, TBA, CK, HBDH, UA, CKMB, K, and Na was 10.16, 42.23, 99.96, 7.94, 500, 45.20, 4.07, and 501.8, respectively. The HI threshold calculated from the equation for LDH was negative, so we did not obtain the HI threshold for LDH.

**Table 2:** Equations produced by curve fits using GraphPad Prism 7 and the HI thresholds calculated from the equations

Analytes	TAE	Functions	R2		Hemolysis Index threshold	Hemoglobin † (g/L)
Aspartate aminotransferase	+20%	Bias% = 13.63 + 0.63*HI‐0.29e‐03*HI^2	0.98	*P* < 0.05	10.16	0.65
Total bile acid	−10%^a^	Bias% = 0.65‐0.26*HI+18.53e‐05*HI^2	0.98	*P* < 0.05	42.23	0.92
Lactate dehydrogenase	+20%	Bias% = 24.04 + 1.47*HI‐91.81e‐05*HI^2	0.98	*P* < 0.05	NA	>0.5
Creatine kinase	+30%	Bias% = −2.03 + 0.41*HI‐0.10e‐02*HI^2 + 1.04e‐06* HI^3	0.99	*P* < 0.05	99.96	1.40
Hydroxybutyrate Dehydrogenase	+30%	Bias% = 18.15 + 1.50*HI‐92.35e‐05*HI^2	0.99	*P* < 0.05	7.94	0.63
Uric acid	−17%	Bias% = −0.06*HI+5.20e‐05*HI^2	0.85	*P* < 0.05	500	4.73
Creatine kinase‐MB	+3 s (11.58 U/L)	Bias = −1.11 + 0.29*HI‐21.21e‐05*HI^2 + 1.75e‐007*HI^3	0.99	*P* < 0.05	45.20	0.94
Potassium	+0.5 mmol/L	Bias = 0.82*log(HI)	0.90	*P* < 0.05	4.07	0.60
Sodium	−4 mmol/L	Bias = −94.42e‐04*HI+0.74	0.89	*P* < 0.05	501.8	4.75

TAE, total allowed error recommended by CLIA’88 regulations; NA, not available. The total allowed error for hydroxybutyrate dehydrogenase was assumed to be 30% according to experience.

a ±10% bias was set as the accepted TAE for the analytes because they are not included in the CLIA’88 regulations. Hemoglobin was estimated from equation HI = 120*Hb‐68, supplement Figure S1.

## 4 DISCUSSIONS

This study investigated hemolytic effects on twenty‐eight analytes using the Advia 2400 chemistry analyzer. We found that AST, TBA, LDH, CK, CKMB, HBDH, GLU, UA, K, and Na were interfered clinically significantly owing to hemolysis of varying degrees represented by HI; we further determined the HI thresholds in eight analytes. Though Shin et al^10^ reported a study involving the verification of hemolysis effects on clinical chemistry results on Advia 2400, the analyzer that used Roche reagents was a different system from ours and did not determine the HI thresholds in the study. In this study, we used the mechanical trauma model to mimic different hemolysis levels because this method is analogous to the mechanical disruption of erythrocytes that frequently occurs during blood collection.^12^, ^13^, ^20^ Giuseppe Lippi et al 21 reported the cell‐free hemoglobin in nonhemolysis samples was <0.5 g/L, which was validated in this study.

In our study, though the hemolysis interference on biochemistry analytes was dependent on the analyzer system, the interference on CK, AST, LDH, and K was consistent with former studies using the Cobas 6000 c501 analyzer^22^ or Roche analyzers.^15^ This confirmed that a common mechanism underlies the observed hemolysis interference. The mechanisms behind the hemolysis interferences include the additive interferences of released intracellular substances (eg, LDH, AST, K, and HBDH) and the chemical interferences when the released substances interacted with the measured analyte (eg, CK and CKMB); 23 it was reported that intracellular adenylate kinase might interfere with the CK assay.^12^ In addition, our results showed that positive hemolysis interferences on CK‐MB activity started to increase at lower HI values compared with CK activity, which was in accordance with Oğuzhan Özcan's study.^24^ The reason may be that the errors from the interfering agents released by hemolysis were amplified by multiplying a constant; this constant parameter is commonly used to calculate the CKMB activity in the assay. We also observed that UA, GLU, Na, and TBA decreased due to hemolysis, which may result from the dilution effects caused by the leakage of intracellular components into the surrounding fluid. However, GLU was less affected in MH than SH, which was also reported in another study.^12^ This phenomenon might be due to the interaction between the spectral interference of the released hemoglobin and the dilution effects owing to the leakage of intracellular components.

We found that the HI thresholds for AST (10.16), HBDH (7.94), and K (4.07) were even lower than the slight hemolysis flag judgment (23 ~ 110) recommended by the manufacturer, which implies that the hemolysis flag is not sensitive enough to detect hemolytic effects in these analytes. Furthermore, this highlights the importance of setting hemolysis warnings based on individual HI thresholds. Lippi et al^16^ reported ADVIA 2400 had a trend toward overestimation of hemolysis compared with other systems, which might, in part, explain this phenomenon. When the bias limit is 20%, the HI calculated from the equation of LDH was negative. This might indicate that the HI threshold was too low to be calculated from the equation. To overcome this problem, more sampling points may be needed to fit a more precise equation.

Some limitations in our study are worth noting. Firstly, hemolysis interference was investigated only at one single concentration level, which was generally normal. On the other hand, the CLSI recommends testing at least two medical decision concentrations.^25^ Secondly, hemolysis produced by aspirating blood through syringe needles does not account for the different hemolysis causes in clinical practice.^1^, ^26^ Finally, the protocol used in the present interference study did not allow us to distinguish the effects of hemoglobin from those of released erythrocytic, leukocytic, and thrombocytic constituents. Future research should focus on a high‐volume and multiple level investigation for the HI thresholds.

In conclusion, this is the first study to our knowledge that investigated HI thresholds using the Advia 2400 analyzer, which extended these HI studies.^10^, ^11^, ^12^, ^13^, ^14^, ^15^, ^16^, ^17^, ^20^, ^21^, ^22^, ^24^ Our results provide HI thresholds for eight analytes (CKMB, CK, K, AST, HBDH, TBA, Na, and UA). These analyses would be inappropriate for reporting when their HI values are higher than the corresponding HI thresholds. The implementation of the assay‐specific HI thresholds can provide an accurate means to identify the extent to which hemolysis interferes with analytes. This would lead to better clinical interpretations and may improve the laboratory test quality by reducing errors associated with hemolysis.

## CONFLICT OF INTEREST

No conflict of interest exists in this manuscript, and the manuscript is approved by all authors for publication. The funding organization(s) played no role in the study design; in the sample collection, analysis, and interpretation of data; in the writing of the report; or in the decision to submit the report for publication.
