# Supplementary material for: Determination of hemolysis index thresholds for biochemical tests on Siemens Advia 2400 chemistry analyzer
Source: J Clin Lab Anal. 2019 Feb 19;33(4):e22856. doi: 10.1002/jcla.22856 (PMC6589729; doi:10.1002/jcla.22856)
Supplement: Supplementary file 1 [file JCLA-33-e22856-s001.docx]

Supplement

Determination of hemolysis index thresholds for biochemical tests on Siemens Advia 2400 chemistry analyzer

**Figure S1:** HI has a nearly linear correlation with free hemoglobin concentrations in plasma (r=0.982, 0<0.05)

Table S1 Model summary and parameter estimates by curve estimation in SPSS and the independent variable is hemolysis index.

|  | **Model Summary** | | | | | | | | | | | | | | | | | | | | | | | | | | | | | **Parameter Estimates** | | | | | | | | | | | | | | | | | | | | | | | | | |
| --- | --- | --- | --- | --- | --- | --- | --- | --- | --- | --- | --- | --- | --- | --- | --- | --- | --- | --- | --- | --- | --- | --- | --- | --- | --- | --- | --- | --- | --- | --- | --- | --- | --- | --- | --- | --- | --- | --- | --- | --- | --- | --- | --- | --- | --- | --- | --- | --- | --- | --- | --- | --- | --- | --- | --- |
| **Equation** | **R Square** | | | | | **F** | | | | | | | | **df1** | | | | | **df2** | | | | | **Sig.** | | | | | | **Constant** | | | | | | | **b1** | | | | | | | | | **b2** | | | | | | | | | **b3** |
| **Dependent Variable: bias% of aspartate aminotransferase (AST) from NH** | | | | | | | | | | | | | | | | | | | | | | | | | | | | | | | | | | | | | | | | | | | | | | | | | | | | | | | |
| Linear | .969 | | | | | 92.644 | | | | | | | | 1 | | | | | 3 | | | | | .002 | | | | | | 27.490 | | | | | | | .434 | | | | | | | | |  | | | | | | | | |  |
| Logarithmic | .836 | | | | | 15.279 | | | | | | | | 1 | | | | | 3 | | | | | .030 | | | | | | -142.632 | | | | | | | 58.108 | | | | | | | | |  | | | | | | | | |  |
| Inverse | .476 | | | | | 2.721 | | | | | | | | 1 | | | | | 3 | | | | | .198 | | | | | | 168.529 | | | | | | | -1217.135 | | | | | | | | |  | | | | | | | | |  |
| Quadratic | .981 | | | | | 51.885 | | | | | | | | 2 | | | | | 2 | | | | | .019 | | | | | | 13.632 | | | | | | | .626 | | | | | | | | | .000 | | | | | | | | |  |
| Cubic | .996 | | | | | 76.591 | | | | | | | | 3 | | | | | 1 | | | | | .084 | | | | | | -3.093 | | | | | | | 1.222 | | | | | | | | | -.003 | | | | | | | | | 3.349E-6 |
| **Dependent Variable: bias% of total bile acid (TBA) from NH** | | | | | | | | | | | | | | | | | | | | | | | | | | | | | | | | | | | | | | | | | | | | | | | | | | | | | | | |
| Linear | | | | .936 | | | | | | 43.561 | | | | | | 1 | | | | | | 3 | | | | | .007 | | | | | | | -8.208 | | | | | | -.141 | | | |  | | | |  | | | | | | | |
| Logarithmic | | | | .848 | | | | | | 16.741 | | | | | | 1 | | | | | | 3 | | | | | .026 | | | | | | | 49.226 | | | | | | -19.346 | | | |  | | | |  | | | | | | | |
| Inverse | | | | .459 | | | | | | 2.549 | | | | | | 1 | | | | | | 3 | | | | | .209 | | | | | | | -54.024 | | | | | | 395.385 | | | |  | | | |  | | | | | | | |
| Quadratic | | | | .982 | | | | | | 54.941 | | | | | | 2 | | | | | | 2 | | | | | .018 | | | | | | | .647 | | | | | | -.263 | | | | .000 | | | |  | | | | | | | |
| Cubic | | | | .992 | | | | | | 40.267 | | | | | | 3 | | | | | | 1 | | | | | .115 | | | | | | | 5.149 | | | | | | -.424 | | | | .001 | | | | -9.015E-7 | | | | | | | |
| **Dependent Variable: bias% of lactate dehydrogenase (LDH) from NH** | | | | | | | | | | | | | | | | | | | | | | | | | | | | | | | | | | | | | | | | | | | | | | | | | | | | | | | |
| Linear | | .952 | | | | | 59.661 | | | | | 1 | | | | | | 3 | | | | | .005 | | | | | | | 67.910 | | | | | | | .866 | | | | | | | | | |  | | | | | | |  | |
| Logarithmic | | .874 | | | | | 20.759 | | | | | 1 | | | | | | 3 | | | | | .020 | | | | | | | -288.250 | | | | | | | 119.560 | | | | | | | | | |  | | | | | | |  | |
| Inverse | | .515 | | | | | 3.189 | | | | | 1 | | | | | | 3 | | | | | .172 | | | | | | | 353.573 | | | | | | | -2549.590 | | | | | | | | | |  | | | | | | |  | |
| Quadratic | | .983 | | | | | 57.638 | | | | | 2 | | | | | | 2 | | | | | .017 | | | | | | | 24.041 | | | | | | | 1.472 | | | | | | | | | | -.001 | | | | | | |  | |
| Cubic | | .997 | | | | | 95.082 | | | | | 3 | | | | | | 1 | | | | | .075 | | | | | | | -8.424 | | | | | | | 2.630 | | | | | | | | | | -.007 | | | | | | | 6.501E-6 | |
| **Dependent Variable: bias% of creatine kinase (CK) from NH** | | | | | | | | | | | | | | | | | | | | | | | | | | | | | | | | | | | | | | | | | | | | | | | | | | | | | | | |
| Linear | | | .987 | | | | | 232.780 | | | | | 1 | | | | | | | 3 | | | | | .001 | | | | | | | 6.381 | | | | | | | .181 | | | | | |  | | | | | | |  | | | |
| Logarithmic | | | .784 | | | | | 10.911 | | | | | 1 | | | | | | | 3 | | | | | .046 | | | | | | | -59.960 | | | | | | | 23.226 | | | | | |  | | | | | | |  | | | |
| Inverse | | | .406 | | | | | 2.051 | | | | | 1 | | | | | | | 3 | | | | | .248 | | | | | | | 63.626 | | | | | | | -464.049 | | | | | |  | | | | | | |  | | | |
| Quadratic | | | .991 | | | | | 112.342 | | | | | 2 | | | | | | | 2 | | | | | .009 | | | | | | | 3.181 | | | | | | | .225 | | | | | | -6.697E-5 | | | | | | |  | | | |
| Cubic | | | .999 | | | | | 646.964 | | | | | 3 | | | | | | | 1 | | | | | .029 | | | | | | | -2.029 | | | | | | | .411 | | | | | | -.001 | | | | | | | 1.043E-6 | | | |
| **Dependent Variable: bias% of hydroxybutyrate dehydrogenase (HBDH) from NH** | | | | | | | | | | | | | | | | | | | | | | | | | | | | | | | | | | | | | | | | | | | | | | | | | | | | | | | |
| Linear | .959 | | | | | 69.354 | | | | | | | | | 1 | | | | | | 3 | | | | .004 | | | | | | 62.277 | | | | | | .889 | | | | | | | | | |  | | | | | |  | | |
| Logarithmic | .863 | | | | | 18.970 | | | | | | | | | 1 | | | | | | 3 | | | | .022 | | | | | | -298.224 | | | | | | 121.642 | | | | | | | | | |  | | | | | |  | | |
| Inverse | .495 | | | | | 2.942 | | | | | | | | | 1 | | | | | | 3 | | | | .185 | | | | | | 353.500 | | | | | | -2557.746 | | | | | | | | | |  | | | | | |  | | |
| Quadratic | .988 | | | | | 84.539 | | | | | | | | | 2 | | | | | | 2 | | | | .012 | | | | | | 18.151 | | | | | | 1.499 | | | | | | | | | | -.001 | | | | | |  | | |
| Cubic | .998 | | | | | 204.803 | | | | | | | | | 3 | | | | | | 1 | | | | .051 | | | | | | -10.476 | | | | | | 2.520 | | | | | | | | | | -.006 | | | | | | 5.733E-6 | | |
| **Dependent Variable: bias% of uric acid (UA) from NH** | | | | | | | | | | | | | | | | | | | | | | | | | | | | | | | | | | | | | | | | | | | | | | | | | | | | | | | |
| Linear | | | | .866 | | | | | | 25.939 | | | | | | 1 | | | | | | 4 | | | | | .007 | | | | | | |  | | | | | | -.034 | | | |  | | | |  | | | | | | | |
| Logarithmic | | | | .696 | | | | | | 9.151 | | | | | | 1 | | | | | | 4 | | | | | .039 | | | | | | |  | | | | | | -2.007 | | | |  | | | |  | | | | | | | |
| Inverse | | | | .000 | | | | | | .002 | | | | | | 1 | | | | | | 4 | | | | | .968 | | | | | | |  | | | | | | -3.784 | | | |  | | | |  | | | | | | | |
| Quadratic | | | | .917 | | | | | | 16.610 | | | | | | 2 | | | | | | 3 | | | | | .024 | | | | | | |  | | | | | | -.063 | | | | 5.197E-5 | | | |  | | | | | | | |
| Cubic | | | | .953 | | | | | | 13.661 | | | | | | 3 | | | | | | 2 | | | | | .069 | | | | | | |  | | | | | | .008 | | | | .000 | | | | 5.034E-7 | | | | | | | |
| **Dependent Variable: bias of creatine kinase-MB (CK-MB) from NH** | | | | | | | | | | | | | | | | | | | | | | | | | | | | | | | | | | | | | | | | | | | | | | | | | | | | | | | |
| Linear | .998 | | | | | 1290.274 | | | | | | | | 1 | | | | | 3 | | | | | .000 | | | | | 30.292 | | | | | | | 2.879 | | | | | | |  | | | | | |  | | | | | | |
| Logarithmic | .738 | | | | | 8.449 | | | | | | | | 1 | | | | | 3 | | | | | .062 | | | | | -965.820 | | | | | | | 356.749 | | | | | | |  | | | | | |  | | | | | | |
| Inverse | .346 | | | | | 1.588 | | | | | | | | 1 | | | | | 3 | | | | | .297 | | | | | 920.342 | | | | | | | -6783.787 | | | | | | |  | | | | | |  | | | | | | |
| Quadratic | .999 | | | | | 1561.817 | | | | | | | | 2 | | | | | 2 | | | | | .001 | | | | | -2.958 | | | | | | | 3.338 | | | | | | | -.001 | | | | | |  | | | | | | |
| Cubic | 1.000 | | | | | 688.577 | | | | | | | | 3 | | | | | 1 | | | | | .028 | | | | | -14.265 | | | | | | | 3.741 | | | | | | | -.003 | | | | | | 2.264E-6 | | | | | | |
| **Dependent Variable: bias of sodium (Na) from NH** | | | | | | | | | | | | | | | | | | | | | | | | | | | | | | | | | | | | | | | | | | | | | | | | | | | | | | | |
| Linear | | | | | .892 | | | | | | 24.793 | | | | | | 1 | | | | | 3 | | | | | | .016 | | | | | | | .738 | | | | | | -.009 | | |  | | | | | | |  | | | | |
| Logarithmic | | | | | .535 | | | | | | 3.453 | | | | | | 1 | | | | | 3 | | | | | | .160 | | | | | | | 3.467 | | | | | | -1.054 | | |  | | | | | | |  | | | | |
| Inverse | | | | | .150 | | | | | | .531 | | | | | | 1 | | | | | 3 | | | | | | .519 | | | | | | | -1.945 | | | | | | 15.509 | | |  | | | | | | |  | | | | |
| Quadratic | | | | | .898 | | | | | | 8.797 | | | | | | 2 | | | | | 2 | | | | | | .102 | | | | | | | .953 | | | | | | -.012 | | | 4.512E-6 | | | | | | |  | | | | |
| Cubic | | | | | .995 | | | | | | 72.075 | | | | | | 3 | | | | | 1 | | | | | | .086 | | | | | | | -.027 | | | | | | .023 | | | .000 | | | | | | | 1.963E-7 | | | | |
| **Dependent Variable: bias of potassium (K) from NH** | | | | | | | | | | | | | | | | | | | | | | | | | | | | | | | | | | | | | | | | | | | | | | | | | | | | | | | |
| Linear | | | .827 | | | | | | 19.114 | | | | | | 1 | | | | | | 4 | | | | | .012 | | | | | | |  | | | | | .005 | | | |  | | | | | | | |  | | | | | |
| Logarithmic | | | .898 | | | | | | 35.256 | | | | | | 1 | | | | | | 4 | | | | | .004 | | | | | | |  | | | | | .358 | | | |  | | | | | | | |  | | | | | |
| Inverse | | | .007 | | | | | | .028 | | | | | | 1 | | | | | | 4 | | | | | .874 | | | | | | |  | | | | | 2.339 | | | |  | | | | | | | |  | | | | | |
| Quadratic | | | .868 | | | | | | 9.824 | | | | | | 2 | | | | | | 3 | | | | | .048 | | | | | | |  | | | | | .009 | | | | -7.299E-6 | | | | | | | |  | | | | | |
| Cubic | | | .946 | | | | | | 11.663 | | | | | | 3 | | | | | | 2 | | | | | .080 | | | | | | |  | | | | | .026 | | | | .000 | | | | | | | | 1.162E-7 | | | | | |

NH, no hemolysis

Figure S2: Scatter plots displaying regression curves with 95%CI curves and formulas for the nine analytes using the bias% and HI data from each group.
